# It is time to have a molecular test for diagnosing *Kingella kingae* infections in the lab!

**DOI:** 10.1128/jcm.01532-25

**Published:** 2025-12-08

**Authors:** Pablo Yagupsky

**Affiliations:** 1 Clinical Microbiology Laboratory, Soroka University Medical Center, Ben-Gurion University of the Negev 26732 , Beer-Sheva, Israel; Endeavor Health, Evanston, Illinois, USA

**Keywords:** *Kingella kingae*, children, skeletal system infections, bacteriological diagnosis, nucleic acid amplification tests

## Abstract

The article by S. J. Oyeniran, A. L. Leber and H. Wang (J Clin Microbiol 63:e00986-25, 2025, https://doi.org/10.1128/jcm.00986-25) describes a novel laboratory-developed diagnostic PCR assay that amplifies the gene encoding *Kingella kingae*’s major outer membrane protein. The test confirmed the prime role of the organism causing skeletal system infections in preschoolers and enabled the administration of targeted antibiotic therapy. It is hoped that a much-needed commercial *K. kingae*-specific molecular test will soon receive FDA clearance to improve the management of pediatric osteoarticular infections.

## COMMENTARY

First characterized by Elizabeth King in the 1960s, *Kingella kingae* was initially considered a rare etiology of human infections (1). Three decades later, the unexpected finding that inoculating synovial fluid aspirates from children with presumptive septic arthritis into blood culture bottles (BCBs) improved the isolation of *K. kingae*, revealing a practical method for recovering the bacterium (1). Systematic use of BCB cultures led to recognition of the organism as an invasive pediatric pathogen, the most common agent of skeletal infections, and a frequent cause of bacteremia, among other conditions, in the preschool population (1, 2).

*K. kingae* is notoriously fastidious, and its growth is inhibited by complement, leukocytes, and antibodies contained in bone and joint exudates. Diluting the tiny biological specimen drawn from a young patient into a large volume of nutrient broth reduces the concentration of these deleterious factors, improving recovery (1). Increasing interest in the organism led to fruitful research that revealed the niche of *K. kingae* in the human body, the epidemiology of upper respiratory tract carriage and invasive infections, and the bacterial population structure (3).

Similar to other *Kingella* species, *K. kingae* is a member of the oropharyngeal and buccal microbiota; its carriage is asymptomatic, and the organism is not isolated from nasopharyngeal cultures (1). *K. kingae* is not detected during the first 6 months of life, likely because maternal antibodies confer immunity to colonization and limited social exposure (4). The prevalence of the organism in the oropharynx increases gradually, reaching a peak of 10–12% during the second year and gradually declines thereafter. *K. kingae* is readily transmitted between susceptible young children through upper respiratory tract secretions and saliva during close contact with a carrier (4). As expected, transmission is more effective during seasonal viral infections and is enhanced among young siblings and playmates (5) and among young daycare attendees, among whom the prevalence exceeds 20% (6). The carriage rate in older children and adults is low (around 1–2%) and transient (7). Outbreaks of *K. kingae* infections in the daycare setting have been reported. They are characterized by concomitant or antecedent viral infections, such as the common cold, hand-foot-and-mouth disease, and herpangina, suggesting that mucosal damage and ulcers facilitate the entry of carried *K. kingae* organisms into the bloodstream (8–11). Colonization is a dynamic process, and new strains are acquired over weeks of continuous carriage, replacing older strains. This turnover suggests the acquisition of strain-specific immunity, which, although it eradicates previously carried organisms, does not prevent colonization by antigenically different strains (4).

*K. kingae* organisms colonizing the oropharynx may penetrate the epithelial surfaces and enter the bloodstream, aided by a potent repeat-in-toxin (RTXA) (12). Once in the bloodstream, the RTX toxin and additional virulence factors, such as a polysaccharide capsule and an exopolysaccharide, ensure bacterial survival by inhibiting opsonin deposition and complement-mediated killing (12–15). *K. kingae* exhibits a predilection for invading joints, bones, the intervertebral discs, and the endocardial tissue. Skeletal system infections, and particularly septic arthritis, are the most common presentations. The incidence of invasive *K. kingae* disease overlaps the age-related prevalence of oropharyngeal colonization, and 95% of infections occur in 6- to 48-month-old children (1).

Whereas in pediatric septic arthritis and osteomyelitis caused by traditional pathogens, blood cultures are positive in 20% and 41% of patients, respectively, reaching 54% in staphylococcal bone infections, the yield in *K. kingae* infections is much lower (16, 17). In a retrospective study of 81 children with culture-proven *K. kingae* skeletal infections, the organism was isolated from the source (bone or joint) culture in 74 (91.4%), from the blood in 6 (7.4%), and from both blood and source in 1 (1.2%), implying that the antecedent bacteremic phase is transient and short-lived (1).

*K. kingae* infections of the skeletal system generally involve the large weight-bearing joints and long bones of the extremities. However, the metacarpophalangeal, sternoclavicular, tarsal, and pelvic joints, as well as the vertebrae, clavicle, tarsal, and metatarsal bones, which are rarely invaded by pyogenic bacteria, are overrepresented in *K. kingae* disease (1). Infections of the spine and sacroiliac joints are difficult to access and aspirate, and the bacteriological diagnosis is particularly problematic. Pediatric orthopedists are frequently unwilling to subject young non-cooperative children to a spine biopsy. In addition to the general anesthesia risks, biopsies may damage the spinal tissues and result in progressive degeneration of the intervertebral disk (18). In a retrospective study, blood cultures were obtained in 62 out of 124 children with spondylodiscitis, with no positive results, and a spinal biopsy was performed in only two patients, and no pathogens were recovered (19).

To overcome the difficulty, a non-invasive surrogate approach has been proposed and implemented. Because pharyngeal colonization is an essential precondition for the development of a *K. kingae* invasive infection, swabbing the oropharyngeal mucosa and performing a sensitive species-specific nucleic acid amplification test (NAAT) has been used as a substitute specimen for diagnosing *K. kingae* infections of the skeletal system (20–22). The exquisite sensitivity of the molecular test confers the approach a high negative predictive value, ruling out the organism as the etiology of the disease. However, because the background carriage rate of *K. kingae* in preschool children is 10% and is twice as high among those attending daycare facilities, the test’s positive predictive value is suboptimal (1).

With the notable exception of endocarditis, the clinical features of invasive *K. kingae* infections are typically subtle, and patients often experience a favorable clinical course, requiring a high index of clinical acumen. Children generally present in good general condition, afebrile or with mild to moderate fever, and with normal or only slightly elevated white blood cell counts and acute-phase reactant levels (1, 23). However, no laboratory inflammation marker, alone or in combination, can reliably discriminate between *K. kingae* joint and bone infections and those caused by other bacteria (1, 24). The only characteristic that consistently separates *K. kingae* skeletal system infections from those caused by gram-positive microorganisms—and *S. aureus* in particular—is the patient’s age: almost all *K. kingae* infections are detected in children aged 6–48 months, whereas other pathogens like *Streptococcus agalactiae* and Enterobacterales primarily affect neonates, or predominate in skeletal system infections of school-aged children and adolescents (*Staphylococcus aureus*, *Streptococcus pyogenes*, *and Neisseria gonorrhoeae*) (24).

An early and accurate diagnosis of *K. kingae* disease has significant therapeutic implications given its distinct antibiotic susceptibility profile. The organism rarely produces penicillinase and remains highly susceptible to cephalosporins and β-lactamase inhibitors (25–28). However, *K. kingae* exhibits high MIC values (MIC_50_: 3 mg/L; MIC_90_: 6 mg/L) for isoxazolyl penicillins, such as oxacillin and cloxacillin, and treatment failure with flucloxacillin has been reported in a child with spondylodiscitis (27, 29). *K. kingae* is intrinsically resistant to vancomycin and clindamycin, antimicrobial drugs empirically administered to children with skeletal infections in regions with a high prevalence of methicillin-resistant *S. aureus* (27). Therefore, timely confirmation of *K. kingae* as the causative agent of the disease can prevent unnecessary administration of broad-spectrum antibiotics and ineffective therapy.

Although the isolation of the bacterium is irrefutable proof of the disease’s etiology, the culture recovery and identification of *K. kingae* take, on average, 2–4 days, and many infections, even when the BCB method is employed, remain bacteriologically unconfirmed (1). The development and implementation of NAAT over the past few years have revolutionized the diagnosis of infectious diseases, particularly those involving the skeletal system. Molecular methods provide rapid, accurate results, confirming the diagnosis, shortening the detection and speciation of pathogens from days to hours, and thus saving unnecessary laboratory and imaging workup. This culture-independent technology improves the detection of fastidious microorganisms and reduces the frustrating fraction of “culture-negative” skeletal system infections. Because molecular methods detect both viable and nonviable organisms, NAATs are less affected by antimicrobial therapy than cultures, enabling diagnosis in patients already receiving antibiotics (30).

The initial method employed “universal primers” that anneal to conserved regions of the 16S rRNA gene in a conventional PCR format, followed by amplicon sequencing and alignment to a curated pathogen database, enabling precise identification. Alternatively, the amplification products were hybridized with pathogen-specific probes. Theoretically, the approach enabled the detection of any bacteria; however, it was time-consuming and prone to contamination. The universal gene amplification assay exhibited a detection threshold for *K. kingae* of 300 colony-forming units (CFUs) per mL, suggesting that the test possesses insufficient sensitivity to detect the low concentration of the organism usually present in synovial fluid specimens (from 11 to 300 CFUs/mL, median 15 CFUs/mL) (1, 31). Despite this limitation, the method improved *K. kingae* detection by a fourfold factor compared to BCB cultures, confirming the bacterium’s prime role in pediatric joint and bone disease (1).

The effect of implementing the broad-spectrum PCR method can be fully appreciated in a Spanish study that assessed the microbiological diagnosis of infectious arthritis across two consecutive periods. During the 7 years from 2002 to 2008, before the use of molecular technology, traditional cultures detected a pathogen in 11 out of 35 (31.4%) children, but not *K. kingae* (32). Between 2009 and 2023, following the use of the molecular assay for joint fluid aspirates and bone exudates, the diagnostic yield increased to 29 out of 46 (63.0%) infections, of which 14 (48.3%) were caused by *K. kingae* (32).

In recent years, the original technology has been superseded by real-time PCR assays targeting *K. kingae*-specific genes, which enable the simultaneous, rapid analysis of multiple specimens, are less labor-intensive and less susceptible to contamination, and offer higher sensitivity. The primary limitation of this strategy is that it amplifies only the pathogen’s DNA sequences targeted by the species-specific primers and probes. Thus, the choice of bacterial target is an informed decision based on clinical and epidemiological considerations and requires specialized expertise. However, due to the prime role of *K. kingae* as a pathogen in the 6- to 48-month age group, the use of a standalone species-specific NAAT is particularly suitable for a preschooler presenting with a suspected joint or bone infection. The most persuasive case for the routine use of a *K. kingae*-specific molecular test for diagnosing joint and bone infections in pediatric patients was presented in a retrospective study involving 217 patients from Switzerland. In addition to cultures, the authors employed a real-time NAAT targeting the *rtx* operon in blood samples, skeletal system specimens, and pharyngeal swabs (2). *K. kingae* was the prime etiologic agent and was detected in 66 out of 138 (47.8%) microbiologically confirmed infections, and in 76 out of 85 (89.4%) children aged 6–48 months (2).

Recently, a *K. kingae*-specific assay (the KING-UX test, manufactured by Progenie Molecular, Valencia, Spain) has been introduced into clinical practice in Europe and successfully employed to establish the diagnosis of septic arthritis in 32 French children aged 7 months to 7 years (33). Another, still experimental, assay developed by Quest Diagnostics in San Juan Capistrano, California, has also shown promising results (34). However, these two species-specific NAATs have not yet been cleared by the Food and Drug Administration (FDA), and no commercial tests are currently available in the United States.

In the absence of a commercial test, over the years, researchers have employed a variety of in-house *K. kingae*-specific NAATs that amplify the *rtxA* and *rtxB* genes of the *rtx* operon, the *groEL* gene (also known as *cpn60*) that encodes the chaperonin 60 protein, and the *mdh* that encodes the malate dehydrogenase enzyme (2, 35, 36). The assays targeting the *rtxA* and *rtxB* genes have a sensitivity of 30 CFU/mL but do not differentiate between *K. kingae* and the RTXA-toxin-producing *K. negevensis* (37). The *groEL*-based assay showed suboptimal sensitivity, whereas the *mdh*-amplifying assay detected 18 variants in the gene’s sequence and was positive in 7 *K*. *kingae* synovial fluid samples that were missed by *groEL* and *rtx* locus NAATs (36). The article by Oyeniran et al., published in this issue of JCM, employed a novel *K. kingae*-specific NAAT that amplifies the gene encoding the major outer membrane protein and does not react with *K. negevensis, K. oralis,* and a variety of invasive pediatric pathogens (38). The study, which comprised 500 (!) USA children with suspected joint and bone infections, demonstrates in a large scale that the European and Israeli experience can be generalized to the other populations: *K. kingae* was the most common agent of joint and bone infections in preschoolers; the disease exhibited a benign clinical presentation and a mild inflammatory response; patients followed an uncomplicated course, and the infection responded to adequate antibiotic therapy, leaving no permanent disabilities (38). The timely ordering of the laboratory-developed NAAT and the early availability of test results enabled identification of the causative pathogen within 24 h, guiding etiology-targeted antibiotic therapy and reducing the need for broad-spectrum antimicrobial drugs.

Until a commercial species-specific test is available, the only authorized molecular tool that amplifies *K. kingae* DNA sequences is the BIOFIRE JI multiplex panel (bioMérieux, Marcy-l'Etoile, France) (39). The multiplex approach eliminates the guesswork of establishing the etiology of skeletal system infections by simultaneously targeting a wide array of potential bone and joint pathogens. This strategy is practical and cost-effective, saving time, laboratory resources, and precious patients' samples. However, multiplex PCR tests may exhibit lower amplification efficiency than single PCR reactions. This diminished sensitivity occurs because multiple primer dimer sets can compete for components such as dNTPs and enzymes. Multiplex PCR also requires a single set of amplification conditions—annealing temperatures, primer concentrations, and cycle parameters—for all included targets, making it challenging to optimize individual sequences (40). The BIOFIRE JI panel targets 31 bacterial species (including *K. kingae*), *Candida albicans*, and eight clinically relevant antibiotic-resistant genes. Most studies assessing the test’s performance have enrolled adult patients with infections of bone and joint implants (41). These difficult-to-diagnose and treat infections can be caused by a wide range of opportunistic and uncommon microorganisms that exhibit overlapping symptoms and signs and have unpredictable antibiotic susceptibility patterns.

In summary, over the last three decades, *K. kingae* has evolved from an obscure and rare cause of human disease to a common pediatric pathogen in preschool children, causing bloodborne infections and affecting the skeletal system in particular. The diagnosis of *K. kingae* infections has been hindered not only by the fastidious nature of the organism and the insensitivity of blood cultures, but also by the unavailability of a commercial molecular test. Developing an “in-house” assay targeting *K. kingae* is an expensive investment that may be cost-prohibitive for many hospitals (42). Nowadays, when the organism is suspected in a young child, pediatricians and orthopedists are compelled to order a standalone *K. kingae* PCR test from a reference laboratory; however, the testing results may take 2–7 days, offsetting many of the advantages of a species-specific test (42). It is hoped that a new and much-needed commercial *K. kingae*-specific real-time PCR test will soon be developed and receive FDA clearance. The test will facilitate the timely diagnosis of the organism available for laboratories that lack homemade molecular tools, curtail the use of broad-spectrum antibiotics, and make a substantial contribution to the management of pediatric skeletal system infections.
